# Tunable chiral magneto-transport through band structure engineering in magnetic topological insulators Mn(Bi_1−*x*_Sb*_x_*)_2_Te_4_

**DOI:** 10.1126/sciadv.adt6084

**Published:** 2025-05-16

**Authors:** Peng Chen, Puyang Huang, Zeyu Li, Jieyi Liu, Qi Yao, Qiang Sun, Ang Li, Xinqi Liu, Yifan Zhang, Xinyu Cai, Jiuming Liu, Liyang Liao, Guanying Yang, Zhongkai Liu, Yumeng Yang, Xiaodong Han, Jin Zou, Thorsten Hesjedal, Zhenhua Qiao, Xufeng Kou

**Affiliations:** ^1^ School of Information Science and Technology, ShanghaiTech University, Shanghai 201210, China.; ^2^ Shanghai Institute of Microsystem and Information Technology, Chinese Academy of Sciences, Shanghai 200050, China.; ^3^ University of Chinese Academy of Science, Beijing 101408, China.; ^4^ International Center for Quantum Design of Functional Materials, CAS Key Laboratory of Strongly-Coupled Quantum Matter Physics, and Department of Physics, University of Science and Technology of China, Anhui 230026, China.; ^5^ Department of Physics, Clarendon Laboratory, University of Oxford, Oxford OX1 3PU, UK.; ^6^ Diamond Light Source, Harwell Science and Innovation Campus, Didcot OX11 0DE, UK.; ^7^ ShanghaiTech Laboratory for Topological Physics, ShanghaiTech University, Shanghai 201210, China.; ^8^ School of Physical Science and Technology, ShanghaiTech University, Shanghai 201210, China.; ^9^ State Key Laboratory of Oral Diseases, National Clinical Research Center for Oral Diseases, West China Hospital of Stomatology, Sichuan University, Chengdu, Sichuan 610041, China.; ^10^ Beijing Key Laboratory of Microstructure and Property of Advanced Materials, Beijing University of Technology, Beijing 100124, China.; ^11^ Institute for Solid State Physics, University of Tokyo, Kashiwa 277-8581, Japan.; ^12^ College of Advanced Interdisciplinary Studies and Nanhu Laser Laboratory, National University of Defense Technology, Changsha, Hunan 410073, China.; ^13^ Department of Materials Science and Engineering, Southern University of Science and Technology, Shenzhen 518055, China.; ^14^ School of Mechanical and Mining Engineering and Centre for Microscopy and Microanalysis, The University of Queensland, QLD 4072, Australia.; ^15^ Institute of Energy Materials Science, University of Shanghai for Science and Technology, Shanghai 200093, China.; ^16^ Hefei National Laboratory, University of Science and Technology of China, Hefei 230088, China.

## Abstract

Berry curvature and spin texture are representative tuning parameters that govern spin-orbit coupling–related physics and are also the foundation for future device applications. Here, we investigate the impact of the Sb-to-Bi ratio on shaping the electronic band structure and its correlated first- and second-harmonic magneto-transport signals in the intrinsic magnetic topological insulator Mn(Bi_1−*x*_Sb*_x_*)_2_Te_4_. First-principles calculations reveal that the introduction of Sb not only triggers a topological phase transition but also changes the integral of the Berry curvature at the shifted Fermi level, which leads to the reversal of the anomalous Hall resistance polarity for Sb fractions *x* > 0.67. Moreover, it also induces the opposite spin splitting of the valence bands compared to the Sb-free host, and the resulting clockwise/counterclockwise spin chirality gives rise to a tunable unidirectional second-harmonic anomalous Hall response. Our findings pave the way for constructing chiral spin-orbitronic devices through band structure engineering.

## Teaser

Engineering Berry curvature and spin texture via band structure design opens an avenue for chiral spintronic applications.

## INTRODUCTION

Spin-orbit coupling (SOC), characterized by the spin Hall effect (*1*–*4*) and the interfacial Rashba effect (*5*, *6*), has exhibited notable potential in various spintronic applications including nonvolatile magneto-resistive random access memory (*7*, *8*), spin logic devices (*9*, *10*), and neuromorphic computing (*11*, *12*). To enable high-performance SOC-based spintronic devices, the key lies in integrating magnetic order and the spin current in an effective manner. For example, in the heavy metal/ferromagnet heterostructures, the spin-orbit torque (SOT) generated from the heavy metal layer can induce spin precession of the magnetic moment in the adjacent ferromagnet layer, thereby fulfilling the magnetization switching function. In this regard, the spin Hall angle, quantifying the efficiency of charge-to-spin conversion, is found to be 0.13 (Pt) (*13*), −0.33 (β-W) (*14*), −0.13 (Ta) (*15*), and −0.28 (Hf) (*16*); meanwhile, the critical switching current density is on the order of 10^6^ A/cm^2^ at room temperature. Compared to heavy metals, topological insulators were predicted to excel at spin current generation due to the spin-momentum locking mechanism of the topologically nontrivial surface states (*17*, *18*). Experimentally, deterministic SOT-driven magnetization switching has been documented in various topological insulator-based magnetic heterostructures with much higher spin Hall angle values (>1) and lower switching current densities (as low as 10^5^ A/cm^2^ at *T* = 298 K and 10^4^ A/cm^2^ at cryogenic temperatures) (*19*–*22*). As a result, the use of topological insulators for constructing energy-efficient devices has propelled the rapid advancement of spin-orbitronics in the past decade (*17*, *18*).

In general, SOC is closely correlated with the electronic band structure of a given material (*5*, *23*). For example, the intrinsic anomalous Hall response originates from the integration of the Berry curvature of each occupied band across the Fermi sea. It reflects interband coherence, contributing to the anomalous velocity, which is closely tied to the Berry phase in momentum space (*24*). In the meantime, the direction of the SOC-induced effective magnetic field is determined by the spin chirality at the Fermi surface (*E*_F_) (*25*, *26*). In this context, Mn(Bi_1−*x*_Sb*_x_*)_2_Te_4_ (MBST), which belongs to the family of the intrinsic magnetic topological insulator MnBi_2_Te_4_ (MBT) (*27*), is created through the substitution of Bi atoms with Sb atoms in the host MBT matrix (*28*). In this material system, with increasing the Sb-to-Bi ratio, the modified Berry curvature distribution leads to a topological phase transition (*28*), and an accompanied Weyl semimetal state may emerge affecting the large negative *c*-axis longitudinal magnetoresistance (*29*). Moreover, introducing Sb is also expected to modify the overall spin texture of the energy band. Accordingly, the MBST system may serve as a promising platform for manipulating the SOC-related effects through band structure engineering.

Here, we report the tuning of the magneto-resistance (MR) and anomalous Hall effect (AHE) responses in five septuple layer (SL) MBST thin films. Both the *x*-dependent magneto-transport results and the density functional theory (DFT) calculations support our conclusions that the reversal of the first-order AHE polarity stems from the opposite Berry curvatures which are dominant for different Sb concentrations. Moreover, the magnetic field and angular-dependent second-harmonic Hall responses also change the sign for *x* > 0.95, triggered by the switching of spin chirality and potential gradient direction during the MnBi_2_Te_4_-to-MnSb_2_Te_4_ transition. Our findings highlight the importance of Berry curvature and spin texture in tailoring the magneto-transport response. These insights will enable the development of chiral SOT-related device applications based on the magnetic topological insulator MBST.

## RESULTS

### Structural properties and phase transition calculations of five SL MBST samples

**Fig. 1.**
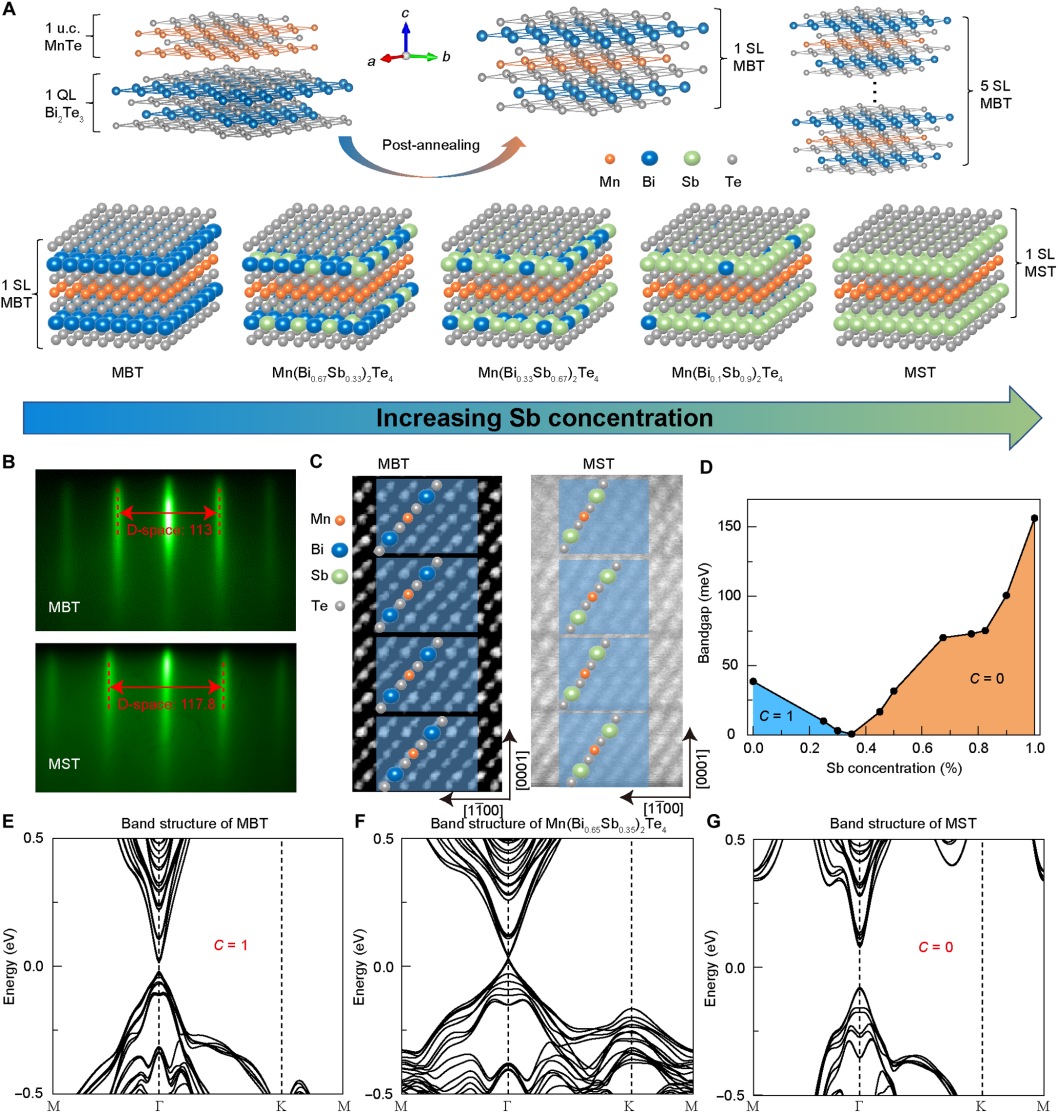
Structural characterizations and DFT calculations of the MBE-grown Mn(Bi_1−*x*_Sb_x_)_2_Te_4_ thin films. (**A**) Schematic of the growth procedure of five SL MBST with varied Sb-to-Bi ratios, starting from a quintuple layer (QL) of Bi_2_Te_3_ and a unit cell (u.c.) of MnTe. (**B**) In situ RHEED patterns of the MBT and MST samples. The sharp streaky patterns sustain during the entire growth. The length of the double arrows changes from 113 (MBT) to 117.8 (MST) reciprocal lattice units, indicating a decrease in the in-plane lattice constant due to the incorporated Sb atoms. (**C**) Cross-sectional HR-STEM image of the MBT and MnSb_2_Te_4_ thin film grown on Al_2_O_3_ (0001) substrates. (**D**) Evolution of the energy gap with Sb content in the MBST system. The closing and reopening of the bandgap *E*_g_ at *x* = 0.35 imply that MBST experiences a topological phase transition from a Chern insulator (*C* = 1) to a normal insulator (*C* = 0). (**E** to **G**) Electronic band structures of the *x* = 0, 0.35, and 1 samples exemplify the topological phase transition in the MBST system.

Experimentally, single-crystalline Mn(Bi_1−*x*_Sb*_x_*)_2_Te_4_ thin films were grown on the 2-inch Al_2_O_3_(0001) substrates by molecular beam epitaxy (MBE). Drawing from our prior work, the septuple-layer structure of MBT can be established by alternating the Bi_2_Te_3_ and MnTe monolayers, followed by a dedicated post-annealing process (*30*). Following the same basic protocol for the MBST sample growth, we incorporated Sb in the (Bi_1−*x*_Sb*_x_*)_2_Te_3_ layer (Fig. 1A). During growth, the structural quality of the films was monitored using in situ reflection high-energy electron diffraction (RHEED). As illustrated in Fig. 1B, the sharp and streaky RHEED patterns of MBT and MnSb_2_Te_4_ (MST) manifest the two-dimensional epitaxial growth mode. With increasing Sb incorporation, the spacing between the first-order RHEED streaks (which is related to the inverse of the *d*-spacing of the direct lattice) gradually enlarges from 113 (MBT) to 117.8 (MST) reciprocal lattice units, suggesting that the in-plane lattice constant of MST is reduced by 4.07% compared to that of MBT (*31*). Consistent with the RHEED data, the Sb concentration *x* within the MBST samples was also quantified using x-ray photoelectron spectroscopy (fig. S1 and table S1). Moreover, the cross-sectional high-resolution scanning transmission electron microscopy (HR-STEM) images in Fig. 1C and fig. S2 (A and B) visualize the well-ordered SL structures of MBT/MST, while the sharp x-ray diffraction patterns of the MBST samples exhibit a series of (00*n*) peaks, with no evidence suggesting the presence of a secondary phase (fig. S2, D to F). In addition, the electronic band structure of MBST (0 ≤ *x* ≤ 1) was investigated by DFT-based first-principles calculations, and the Sb-induced topological phase transition is observed in Fig. 1D. Specifically, a nontrivial topological surface state with a negative *E*_g_, induced by the inverted band, appears in MBT (Fig. 1E) (*28*). With the increase of the Sb content, the inverted bandgap gradually shrinks (i.e., due to the relatively weakened SOC of the Sb atoms) (*28*) until it is closed at *x* = 0.35 (i.e., the gapless band structure in Fig. 1F), which indicates the completion of topological phase transition from *C* = 1 to *C* = 0 (where *C* is the topological Chern number) (*28*, *32*). With further increasing the Sb-to-Bi ratio, the MBST (*x* > 0.35) system maintains the topologically trivial state with a positive energy gap (Fig. 1G) (*28*, *32*). Therefore, our comprehensive materials characterization results and DFT calculations confirm the high quality of the MBE-grown MBST samples and the tunability of the band structure, which makes them perfectly suited for our systematic doping study.

### AHE and AHC polarity reversals induced by tunable Berry curvature

**Fig. 2.**
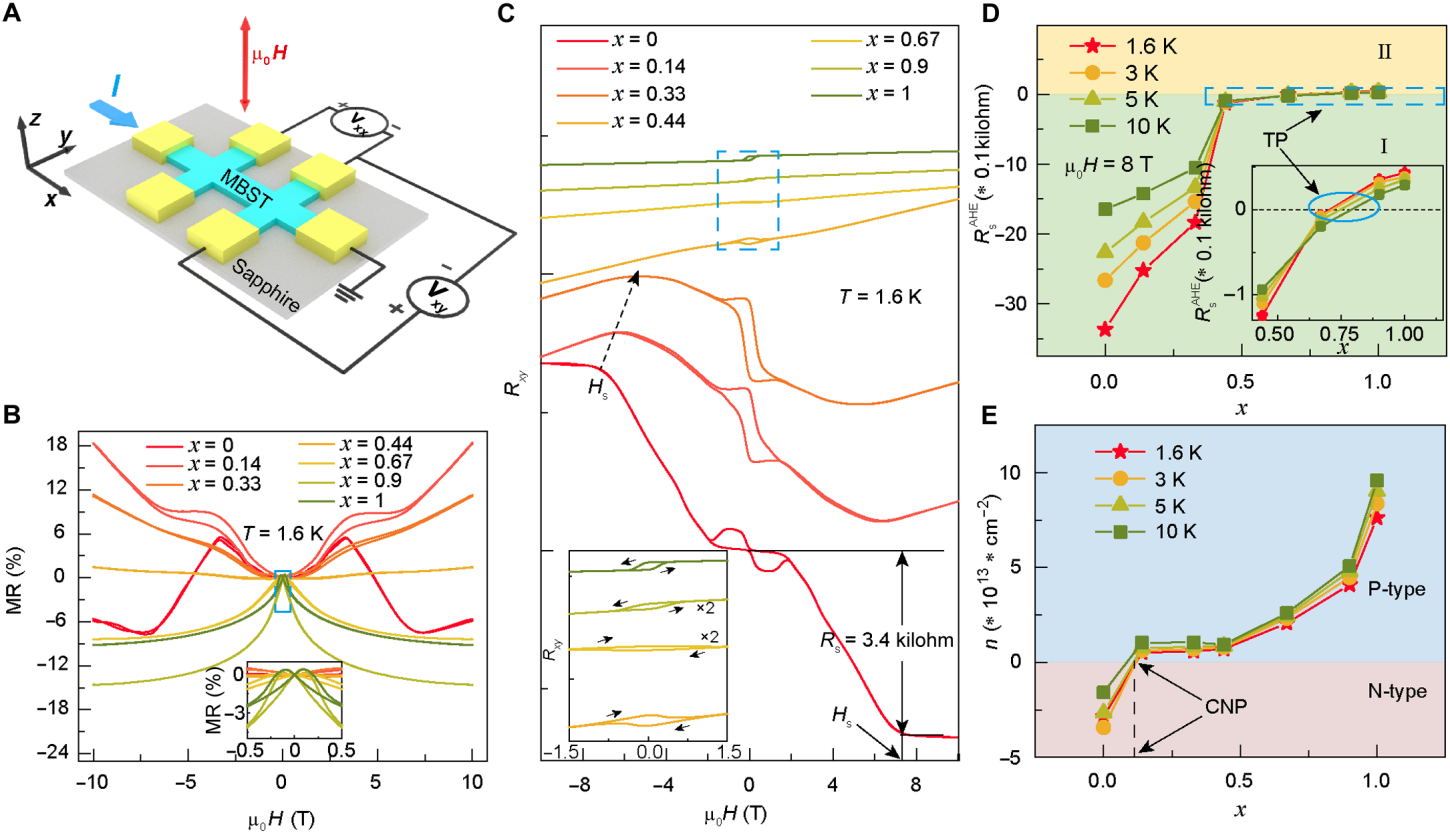
MR and AHE in five SL Mn(Bi_1−*x*_Sb_x_)_2_Te_4_ thin films with *x* = 0, 0.14, 0.33, 0.44, 0.67, 0.9, and 1. (**A**) Schematic of the six-terminal Hall bar device and the magneto-transport measurement setup. The input current *I* and magnetic field μ_0_*H* are applied along the *x* and *z* axes, and the longitudinal (*V_xx_*) and transverse (*V_xy_*) voltages are recorded along the *x* and *y* directions, respectively. (**B**) Magnetic field–dependent MR data at *T* = 1.6 K. The overall MR curve gradually changes from the antiferromagnetic-type giant MR contour to the ferromagnet-like double-split butterfly line shape with the increase of the Sb content (inset). (**C**) Low-temperature anomalous Hall resistance of the MBST samples. With increasing Sb concentration, the saturation field *H*_s_ successively reduces and the AHE loop polarity changes when *x* > 0.67 (inset). The hybrid AHE observed in the MBT thin film may be caused by native antisite defects and/or random stacking order formed during sample growth. (**D**) Temperature-dependent saturated AHE resistance $R_{s}^{\text{AHE}}$ (μ_0_H = 8 T) of the five SL MBST thin films, where the sign of $R_{s}^{\text{AHE}}$ changes from negative to positive at *x* ~ 0.67 (inset) and the “TP” means transition point. (**E**) Carrier density as a function of Sb content *x* in the MBST samples. The charge neutral point (CNP) is located *x* ~ 0.14.

Subsequently, a set of five SL MBST-based micrometer-sized six-terminal Hall bar devices was fabricated using standard photolithography and ion beam etching, and their magnetic/electrical properties were investigated by magneto-transport measurements, where the *x* axis was defined as the current conduction direction and the magnetic field was applied along the *z* axis (Fig. 2A). As depicted in Fig. 2B, the overall MR responses display two distinctive features. First, the low-field MR curve undergoes a positive-to-negative transition with increasing Sb content (*x*), which should be associated with the topological phase transition (*29*). Furthermore, the antiferromagnetic hump-like giant MR profile of the MBT thin film gradually transits into a ferromagnetic-like double-split butterfly MR slope (inset of Fig. 2B) in samples with *x* ≥ 0.67. This evolution highlights the role of Sb atoms in modulating the interlayer magnetic coupling within the MBST samples (*29*). Likewise, the characteristic saturation field (*H*_S_) in reference to the measured anomalous Hall resistance (*R*_S_) monotonically decreases with *x* (i.e., indicated by the dashed arrow in Fig. 2C), suggesting a weakening trend of interlayer antiferromagnetic coupling. Notably, with further increasing the Sb-to-Bi ratio, the polarity of the AHE hysteresis loop is reversed when *x* > 0.67 (inset of Fig. 2C). Correspondingly, the saturated anomalous Hall resistance (i.e., after subtracting the ordinary Hall component) at μ_0_*H* = 8 T also changes from negative (*x* ≤ 0.67, area I) to positive (*x* ≥ 0.9, area II) when 1.6 K ≤ *T* ≤ 10 K (Fig. 2D). Here, it should be noted that the charge neutrality points of the MBST samples extracted from the carrier density (*n*) chart are found to be located at *x* ~ 0.14 in the low-temperature region (Fig. 2E). With the further increase of the Sb content, the Fermi level progressively shifts toward the valence band below the Dirac point, which mainly determines the *R_xy_* slope in the high-field region (i.e., yet it does not contribute to the AHE polarity change). Therefore, the systematic MR and *R_xy_* results reveal the critical role of the band structure (i.e., by controlling the Sb-to-Bi ratio) in manipulating the magneto-transport behavior and magnetic coupling in the MBST system.

**Fig. 3.**
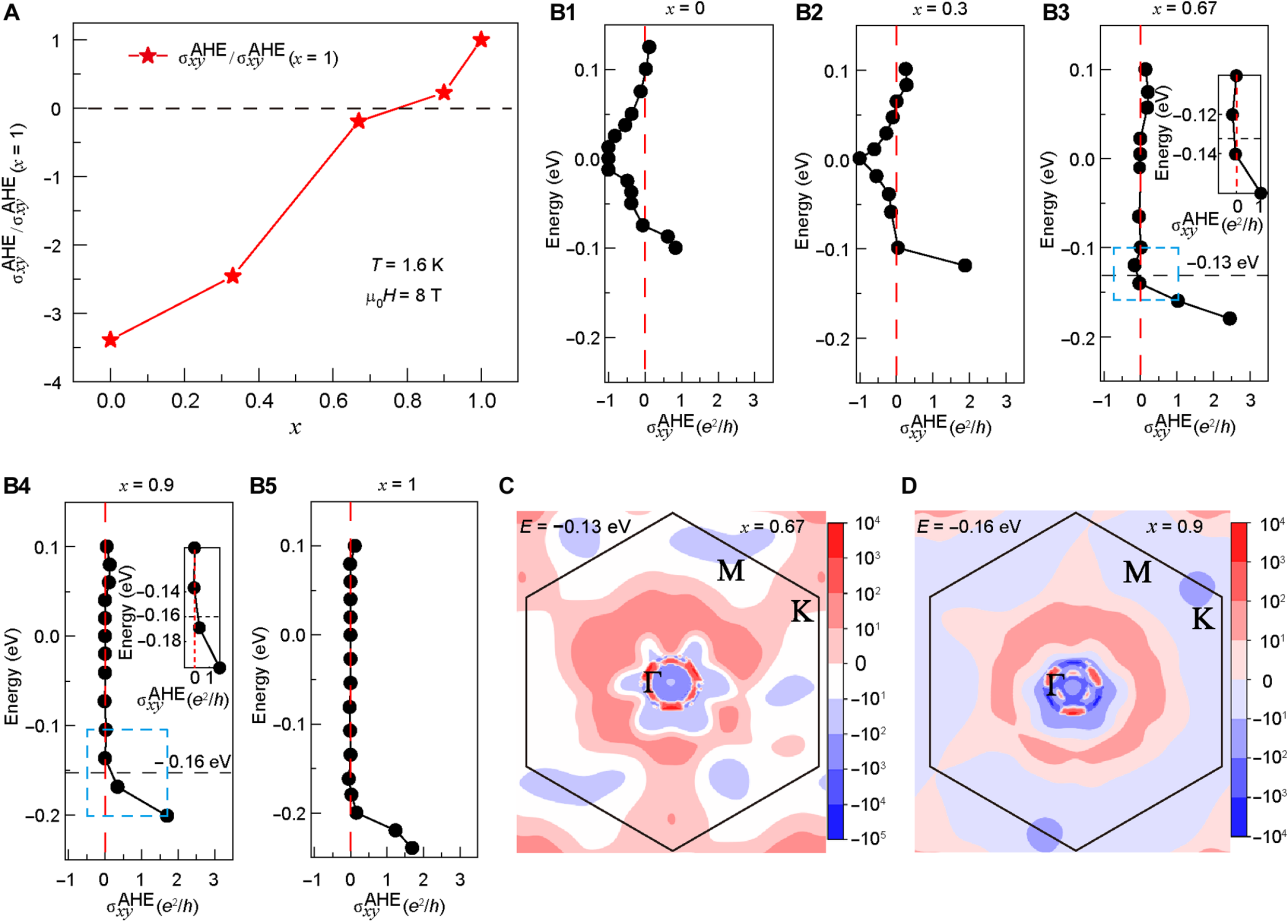
Tailoring the AHC and Berry curvature via the Sb-to-Bi ratio engineering. (**A**) The normalized AHC displays a negative-to-positive transition in the five SL MBST samples at *T* = 1.6 K. (**B1** to **B5**) The calculated intrinsic anomalous conductance as a function of the chemical potential. Negative AHC pockets are observed in the *x* = 0, 0.3, and 0.67 cases, whereas only positive AHC values are present in the *x* = 0.9 and 1 samples. The inset figures of (B3) and (B4) display the zoomed-in regions indicated by the light blue dashed boxes. (**C** and **D**) Berry curvature distributions at −0.13 eV (C) and −0.16 eV (D) corresponding to negative [inset of (B3)] and positive [inset of (B4)] AHC values, which visualize the competition between opposite Berry curvature components in the five SL MBST samples (*x* = 0.67 and 0.9). The integral of the Berry curvature in the *k*_x_-*k*_y_ plane determines the intrinsic AHC polarity.

To understand the fundamental origin of the AHE in the five SL-MBST samples, we plotted the normalized anomalous Hall conductance (AHC) $\sigma_{\mathrm{xy}}^{\text{AHE}}/\sigma_{\mathrm{xy}}^{\text{AHE}}\left(x=1\right)$ [where $\sigma_{\mathrm{xy}}^{\text{AHE}}=R_{\mathrm{xy}}/\left(R_{\mathrm{xx}}^{2}+R_{\mathrm{xy}}^{2}\right)$] as a function of the Sb concentration in Fig. 3A. As can be seen, the signs of the AHC of the *x* = 0.45 and 0.67 samples are opposite to those of the *x* = 0.9 and 1 counterparts. Accompanying this dataset, the intrinsic Hall conductance for different *x* values was calculated as the integral of the Berry curvature of all occupied states within the Brillouin zone (*24*, *33*)

$$\begin{array}{c} \sigma_{\mathrm{xy}}^{\text{AHE}}=-\frac{2\pi e^{2}}{h}\sum_{a}\int \frac{dk}{{\left(2\pi \right)}^{3}}f_{a}\left(k\right)\Omega_{a}\left(k\right)\\ \Omega_{a}\left(k\right)=\frac{2ih^{2}}{{\left(2\pi \right)}^{2}}\sum_{a\neq b}\frac{\left\langle k,a|{\hat{\upsilon }}_{x}|k,b\right\rangle \left\langle k,b|{\hat{\upsilon }}_{y}|k,a\right\rangle }{{\left[E_{a}\left(k\right)-E_{b}\left(k\right)\right]}^{2}} \end{array}$$ (1)

where *e* is the electron charge, *h* is the Planck’s constant, and $f_{a}\left(k\right)$ denotes the Fermi-Dirac distribution of the occupied states. Regarding the Berry curvature $\Omega_{a}\left(k\right)$ in ***k*** space, ${\hat{\upsilon }}_{x}=\frac{2\pi i}{h}\left[\hat{H},{\hat{r}}_{x}\right]$ and ${\hat{\upsilon }}_{y}=\frac{2\mathrm{\pi i}}{h}\left[\hat{H},{\hat{r}}_{y}\right]$ represent the velocity operators along the *x* and *y* directions, while ⟨k,a∣ (⟨k,b∣) and $E_{a}\left(k\right)$ []$E_{b}\left(k\right)$ denote the eigenvectors and eigenvalues of the Hamiltonian $\hat{H}$, respectively. Figure 3(B1 to B5) summarizes the calculated intrinsic $\sigma_{\mathrm{xy}}^{\text{AHE}}$ as a function of the chemical potential in the Mn(Bi_1−*x*_Sb*_x_*)_2_Te_4_ samples (*x* = 0, 0.3, 0.67, 0.9, and 1). It is seen that negative AHC pockets are well-developed for the *x* = 0, 0.3, and 0.67 cases, yet they progressively diminish as the Sb content increases. When *x* > 0.67, only positive AHC values are obtained in the $\sigma_{\mathrm{xy}}^{\text{AHE}}-E$ spectra. These DFT simulation results suggest that the intrinsic $\sigma_{\mathrm{xy}}^{\text{AHE}}$ experiences a negative-to-positive transition as more Bi atoms are substituted by Sb, qualitatively consistent with our experimental data [i.e., to quantitatively reconstruct Fig. 3A, extrinsic contributions from side-jump and skew scatterings need to be considered in a more comprehensive AHC model for future studies (*24*)]. In alignment with such a critical AHC transition, the corresponding Berry curvature distributions of the two MBST samples (*x* = 0.67 and 0.9) within the *k_x_*-*k_y_* plane are provided in Fig. 3 (C and D). Comparing with the host MBT framework (fig. S3), we can see that while both MBST maps display a threefold symmetry-breaking feature, the amplitude of $\Omega_{a}\left(k\right)$ at each occupied (*k_x_*, *k_y_*) state exhibits a strong dependence on the Sb-to-Bi ratio, namely, the positive (negative) Berry curvature components dominate the *x* = 0.67 (0.9) sample, hence resulting in an overall negative (positive) AHC [i.e., it is noted from Eq. 1 that the intrinsic $\sigma_{\mathrm{xy}}^{\text{AHE}}$ is negatively associated with the Berry curvature $\Omega_{a}\left(k\right)$ according to conventional definition (*24*)]. On this basis, the agreement between experimental and theoretical results confirms that the *x*-tailored Berry curvature can induce the sign reversal of the first-order anomalous Hall response in MBST thin films.

### Tailoring second-harmonic polarity through chiral spin texture

**Fig. 4.**
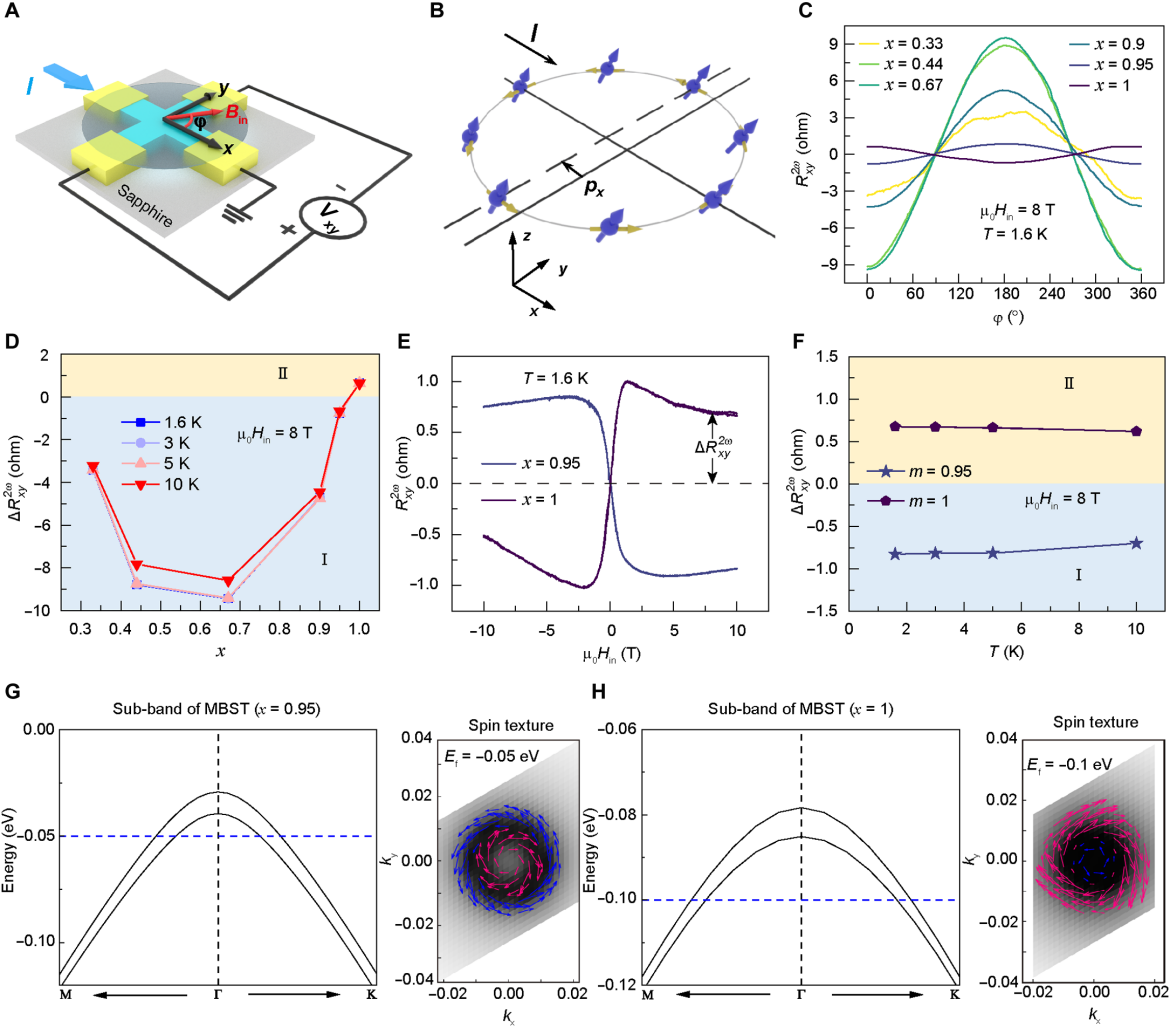
Phase modulation of the unidirectional second-harmonic anomalous Hall resistance in the five SL MBST thin films. (**A**) Schematic of the cross-bar device structure and the angular-dependent second-harmonic measurement setup. The rotation angle is defined as the angle between the applied *I* and in-plane magnetic field μ_0_*H*_in_. (**B**) Illustration of the nonequilibrium spin accumulations generated along the *k_x_* direction by the applied current. (**C**) Angular-dependent plots of $R_{\mathrm{xy}}^{2\omega }$ for the MBST samples (*x* = 0.33, 0.44, 0.67, 0.9, 0.95, and 1) at *T* = 1.6 K and **|**μ_0_*H*_in_**|** = 8 T. All sinusoidal $R_{\mathrm{xy}}^{2\omega }\left(\varphi \right)$ curves have the same period of 360°, yet a peak-to-valley transition occurs in the MST sample (*x* = 1). (**D**) Temperature-dependent $∆R_{\mathrm{xy}}^{2\omega }=\left[R_{\mathrm{xy}}^{2\omega }\left(0{}^{\circ}\right)-R_{\mathrm{xy}}^{2\omega }\left(180{}^{\circ}\right)\right]/2$ extracted from the measured second-harmonic transport data, which confirms that the reversal of the $R_{\mathrm{xy}}^{2\omega }\left(\varphi \right)$ curve is not caused by the temperature variation. (**E**) Magnetic field–dependent second harmonic signals of the Mn(Bi_0.05_Sb_0.95_)_2_Te_4_ and MST samples at *T* = 1.6 K. Both current and in-plane magnetic field are applied along the *x* axis. (**F**) $∆R_{\mathrm{xy}}^{2\omega }-T$ data from 1.6 to 10 K. (**G** and **H**) DFT-simulated valence band structures and corresponding spin texture mapping of the *x* = 0.95 MBST (G) and *x* = 1 MST (H) samples.

In addition to the AHE, the second-harmonic magneto-transport signals of the MBST system were also investigated. Accordingly, the same set of five SL MBST samples (0.33 ≤ *x* ≤ 1) were fabricated into micrometer-size cross-bar devices, and Fig. 4A exemplifies the experimental setup of the angular-dependent second-harmonic measurement, in which the input current [*I* = *I*_0_ sin(ω*t*), *I*_0_ = 100 μA, ω/2π = 31.3 Hz] was applied along the *x* axis, and an external in-plane magnetic field of |μ_0_*H*_in_| = 8 T was successively rotated within the *xy* plane (i.e., the angle between μ_0_*H*_in_ and *x* axis is defined as φ). As illustrated in Fig. 4B, the effective spin-orbit field endowed by the intrinsic SOC can effectively convert the charge current into a nonequilibrium spin current whose scattering is affected by the magnetic moment of the MBST system (*34*). Under such circumstances, the nonreciprocal charge transport would arise where the second-harmonic magneto-transport signals are odd with onefold symmetry under the reversal of either the charge current or the magnetic field (*26*). In agreement with the theoretical expectation (*35*–*37*), an unidirectional second-harmonic anomalous Hall resistance was observed in our five SL-MBST samples (0.33 ≤ *x* ≤ 1), where the measured second-harmonic Hall resistances $R_{\mathrm{xy}}^{2\omega }\left(\varphi \right)$ curves all exhibit the sinusoidal dependence in reference to the rotation angle φ with the same period of 360° at *T* = 1.6 K, yet their relevant peak positions shift from φ = 180° (*x* = 0.33, 0.44, 0.67, 0.9, and 0.95) to φ = 0° (*x* = 1), as shown in Fig. 4C. Concurrently, the high-field amplitudes $∆R_{\mathrm{xy}}^{2\omega }=\left[R_{xy}^{2\omega }\left(0{}^{\circ}\right)-R_{xy}^{2\omega }\left(180{}^{\circ}\right)\right]$/2 of the five SL MBST samples are found to maintain as negative or positive values regardless of the temperature variation (Fig. 4D). Such a different angular-dependent second-harmonic Hall response is also manifested in Fig. 4E, where the in-plane magnetic field–dependent $R_{\mathrm{xy}}^{2\omega }$ curves of the Mn(Bi_0.05_Sb_0.95_)_2_Te_4_ and MST thin films show opposite polarities under the same parallel and antiparallel (φ = 0° and 180°) μ_0_*H*_in_-*I* configurations. This unique feature can be observed in the low-temperature region (e.g., 1.6 K ≤ *T* ≤ 10 K), as shown in Fig. 4F.

Given that the polarity of the second-harmonic signal ($R_{\mathrm{xy}}^{2\omega }$) is closely correlated with the spin texture of the magnetic MBST thin film (i.e., which determines the polarization of the spin current within the conduction channel) (*38*, *39*), the observed peak/valley transition between the *x* = 0.95 and 1 samples indicates the reversal of the spin chirality direction. To understand the difference between the *p*-type *x* = 0.95 and *x* = 1 samples, their valence band (*E*_V_) structures and the spin textures projected onto the *k_x_*-*k_y_* plane were subsequently calculated by first-principles calculations. As shown in Fig. 4 (G and H), the presence of SOC gives rise to the band splitting in the MBST system, and the resulting two sub-bands contain opposite spin chirality. It is identified that the spin direction of the outer sub-band for the *x* = 0.95 sample (counterclockwise blue arrows) is opposite to that of the *x* = 1 one (clockwise red arrows). Consequently, the overall dominant counterclockwise (clockwise) spin texture (i.e., which is the integration of the in-plane spin components from *E*_V_ to *E*_F_) results in the accumulation of a nonequilibrium spin polarization along the −*y* (+*y*)-direction of the five SL-Mn(Bi_0.05_Sb_0.95_)_2_Te_4_ (MST) sample, which in turn is responsible for the appearance of the φ = 180° (φ = 0°) peak position of the $R_{\mathrm{xy}}^{2\omega }\left(\varphi \right)$ curve as well as the negative (positive) $R_{\mathrm{xy}}^{2\omega }\left(0^{{}^{\circ}}\right)$ at |μ_0_*H*_in_| = 8 T. In addition, our simulation results also reveal that the breaking of the periodic lattice condition along the *z* direction can introduce a surface potential at the top MBST surface, and the corresponding potential gradients (∇*V*) are found to be opposite in *x* = 0.95 and 1 cases (fig. S4). Considering that the effective spin-orbit field direction is given by $B_{\text{SO}}\propto \sigma \cdot \left(p\times \nabla V\right)$ (i.e., where σ and ***p*** represent the Pauli spin matrix vector and the momentum operator, respectively), the spin states of the top surface states are thereafter polarized toward the −*y*-axis (Mn(Bi_0.05_Sb_0.95_)_2_Te_4_) and +*y*-axis (MST). In other words, both the opposite spin chirality and potential gradient directions support the possible physical origins of the second-harmonic $R_{\mathrm{xy}}^{2\omega }$ polarity found in our measurement datasets.

## DISCUSSION

In conclusion, we have demonstrated the precise control over the electronic band structure of MBST through systematic adjustment of the Sb-to-Bi ratio. This manipulation enables the tuning of the Berry curvature and spin texture at the Fermi level, which tailors both first-and second-harmonic magneto-transport responses in terms of MR line shape and polarity. The intrinsic interplay between SOC and spin/magnetic orders allows for spin polarization switching via effective band engineering, which may facilitate the further design of SOT-based devices with customizable chirality. With further explorations of the topological features embedded in the host MBT family matrix [e.g., MBST, MBT(Bi_2_Te_3_)*_n_*, and MBST((Bi_1−*x*_Sb*_x_*)_2_Te_3_)*_n_*], our results open up an avenue for the pursuit of energy-efficient topological spintronic applications.

## METHODS

### Sample growth and characterizations

Following our previous optimized growth recipe (*30*), the MBST samples (*x* = 0, 0.14, 0.33, 0.44, 0.67, 0.9, 0.95, and 1) were grown on Al_2_O_3_ (0001) substrates by MBE at a pressure of 1 × 10^−8^ Pa. The Al_2_O_3_ substrate was pre-annealed at 570°C before the sample growth. Next, high-purity Mn (99.9998%), Bi (99.99999%), Sb (99.99999%), and Te (99.99999%) atoms were coevaporated from standard Knudsen cells and cracker cells, and the ratio between the different elements (e.g., Bi and Sb) was validated using a beam flux monitor. Before obtaining the first MBST SL, monolayers of (Bi_1−*x*_Sb*_x_*)_2_Te_3_ and MnTe need to be deposited sequentially at 200° and 370°C, respectively, followed by a moderate post-annealing at 390°C. Meanwhile, the growth process was monitored by in situ RHEED. In addition, x-ray diffraction and x-ray photoelectron spectroscopy were performed to examine the crystal structure as well as to calibrate the ratio of Bi and Sb of the grown samples.

### Device fabrication

The devices investigated in this work were obtained by a standard nanofabrication process. The MBE-grown five SL MBST thin films were firstly capped with a 1.4-μm-thick layer of photoresist before being exposed using a maskless photolithography system (MLA150). The micrometer-sized, six-terminal Hall bar and cross-bar patterns were defined by ion beam etching. Last, 160-nm-thick Ti/Au electrodes were deposited using an e-beam evaporator. All the processes were carried out in a cleanroom with International Organization for Standardization 5 (ISO-5) and ISO-6 level conditions.

### Transport measurement

The magneto-transport measurements of the MBST–based devices were performed using a He^4^ refrigerator (Oxford Teslatron PT system), which provides a base temperature of as low as 1.5 K and the magnetic field up to ±14 T. Subsequently, standard lock-in measurements were performed, where the current amplitude was fixed at 1 μA (first-harmonic Hall measurements) and 100 μA (second-harmonic Hall measurements), respectively, and the lock-in frequency was modulated from 1 to 1000 Hz. As illustrated in Fig. 2A, the current conduction direction was defined as the *x* axis, following the standard convention for six-terminal Hall bar devices, and the longitudinal (*V_xx_*) and transverse (*V_xy_*) voltages are recorded along the *x* and *y* directions during the magneto-transport measurements, respectively. The *x* and *y* directions are independent of the crystal orientation (see section S5). Both the first- and second-harmonic voltage signals were recorded simultaneously by SR-830 lock-in amplifiers (see section S6).

### First-principles calculations

We used the projected augmented wave method as implemented in the VASP package (*40*) and generalized gradient approximation exchange-correlation potential in the calculations (*41*). A 2 × 2 supercell was built to simulate the properties of MBST with different Sb doping concentrations, and the 3*d* states of Mn were treated with the GGA + U approach with *U* = 5.0 eV (*42*). Meanwhile, the kinetic cutoff energy of the plane wave was set up to 350 eV. Subsequently, the Brillouin zone was sampled with a Γ-centered 4 × 4 × 1 grid based on the scheme proposed by Monkhorst and Pack (*43*), a vacuum buffer space over 18 Å was included to prevent interaction between adjacent slabs, and the convergence criterion was set to 10^−5^ eV for energy in optimization and self-consistent calculations, respectively. Besides, the Hellmann-Feynman force tolerance criterion for convergence was 0.01 eV/Å, and the DFT-D3 method was adopted to describe the van der Waals force of the layered MBST (*44*). The maximally localized Wannier functions were constructed to calculate the topological-related properties and spin texture (*45*, *46*).

## Supplementary Materials

### This PDF file includes:

Supplementary Text Figs. S1 to S5 Table S1 References
