# Supplementary material for: Tunable chiral magneto-transport through band structure engineering in magnetic topological insulators Mn(Bi1−xSbx)2Te4
Source: Sci Adv. 2025 May 16;11(20):eadt6084. doi: 10.1126/sciadv.adt6084 (PMC12083519; doi:10.1126/sciadv.adt6084)
Supplement: Supplementary file 1 — Supplementary Text Figs. S1 to S5 Table S1 References [file sciadv.adt6084_sm.pdf]

Science Advances
AAAS

Supplementary Materials for

Tunable chiral magneto-transport through band structure engineering in magnetic topological insulators $Mn(Bi_{1-x}Sb_x)_2Te_4$

Peng Chen *et al.*

Corresponding author: Xufeng Kou, kouxf@shanghaitech.edu.cn; Zhenhua Qiao, qiao@ustc.edu.cn; Thorsten Hesjedal, thorsten.hesjedal@physics.ox.ac.uk

Sci. Adv. **11**, eadt6084 (2025)
DOI: 10.1126/sciadv.adt6084

This PDF file includes:

Supplementary Text
Figs. S1 to S5
Table S1
References

Supplementary Text

Section 1. XPS measurements of the MBE-grown 5 SL $Mn(Bi_{1-x}Sb_x)_2Te_4$ thin films.

To calibrate the Sb-to-Bi ratio in our MBE-grown 5 SL $Mn(Bi_{1-x}Sb_x)_2Te_4$ (MBST) thin films, high-resolution X-ray photoelectron spectroscopy (XPS) measurements were conducted after sample growth. As shown in Fig. S1, the characteristic main peaks (Bi:4*f* 5/2 and 7/2, Sb:3*d* 3/2 and 5/2) are clearly observed in the x = 0.67, 0.9, and 0.95 samples, which are attributed to Bi-Te and Sb-Te bonds. Further, two pairs of additional peaks are indicative of the presence of the Bi-O and Sb-O bonds which are possibly due to the surface oxidation(*47, 48*). Moreover, by integrating the area of the Bi and Sb XPS spectra, the Sb-to-Bi ratios were quantified, which are in agreement with the results obtained using the *in-situ* beam flux monitor (BFM), as summarized in Table S1. Therefore, our XPS measurements demonstrate that the elemental composition of the MBST samples can be precisely controlled.

Section 2. TEM and XRD characterizations of the 5 SL MBST thin films.

In order to quantitatively investigate the sample quality of our MBE-grown $Mn(Bi_{1-x}Sb_x)_2Te_4$ samples, we performed high-resolution scanning transmission electron microscopy (HR-STEM). As shown in Figs. S2A-B, highly-ordered septuple-layer structures are clearly visible in both $MnBi_2Te_4$ (MBT) and $MnSb_2Te_4$ (MST) samples along the [0001] direction. Besides, the Bi and Sb atoms are found to be uniformly distributed within the $Mn(Bi_{0.1}Sb_{0.9})_2Te_4$ sample, as validated by the energy-dispersive X-ray spectroscopy (EDS) mappings in Fig. S2C.

On the other hand, it is known that the introduction of Sb will modify the crystal structure of the $MnBi_2Te_4$ host matrix. Accordingly, we have performed X-ray diffraction (XRD) measurements (using a Cu $K\alpha$ source and a parallel plate collimator) to investigate the MBE-grown MBST thin films. From the XRD spectra of the 5 SL MBT and MST samples displayed in Figs. S2D-E, it is clear that only the series of (00*n*) peaks are present, without signs of any other secondary phases. Figure S2F plots the shifts ($\Delta 2\theta$) of the Al_2O_3(0006), MBST(0012), and MBST(0024) peaks as a function of the Sb content (x). In contrast to the constant Al_2O_3(0006) peak, we find that both the MBST(0012) and MBST(0024) peak positions monotonically shift to

lower diffraction angles with the increase of the Sb content, hence implying a reduced in-plane lattice constant as more Bi sites are substituted by Sb atoms (*i.e.*, rather than the formation of MST patches within the MBT matrix).

Section 3. Intrinsic anomalous Hall conductance and Berry curvature mapping of MBT.

Figure S3 shows the intrinsic anomalous Hall conductance (AHC) and its corresponding Berry curvature mapping of a 5 SL MBT thin film obtained from density functional theory (DFT) calculations. Consistent with other reports(*49, 50*), a quantized AHC of $\sigma_{xy}^{\mathrm{AHE}} = -e^2/h$ is observed at E = 0 eV. According to Eq. (1) in the main text, such a negative AHC means that positive Berry curvature components dominate in the Brillouin zone, as shown in Fig. S3B. Note that in the absence of Sb substitution, the crystal structure of MBT maintains a three-fold symmetry. As a result, the corresponding Berry curvature distribution also exhibits a three-fold symmetry within the k_x–k_y plane. On the other hand, however, we need to point out that it is difficult to quantity the real AHC value as well as precisely locate the Fermi level position from the DFT calculations due to the following limitations: (1) the base temperature was assumed at T = 0 K during our calculations; (2) the calculated intrinsic $\sigma_{xy}^{\mathrm{AHE}}$data points are discrete within the step size (*e.g.* 20~33 meV); and (3) extrinsic contributions from side-jump and skew scatterings were not considered*(24, 51)*.

Section 4. Spatial electrostatic potential distributions of two 5 SL MBST (*x* = 0.95 and 1) samples.

Given that $\boldsymbol{B}_{\mathbf{SO}} \propto \boldsymbol{\sigma} \cdot (\boldsymbol{p} \times \nabla V)$, where $\boldsymbol{\sigma}$ and $\boldsymbol{p}$ represent the Pauli spin matrix-vector and the momentum operator, respectively, the direction of the effective spin-orbit coupling field B_{SO} is determined by the gradient of the electrostatic potential ∇V. From the DFT calculations shown in Fig. S4, it can be seen that the gradient of the electrostatic potential at the top surface is negative for the $Mn(Bi_{0.05}Sb_{0.95})_2Te_4$ sample, whereas it becomes positive for the MST sample. Consequently, the spin states of the top surface states are thereafter polarized towards the –y-axis ($Mn(Bi_{0.05}Sb_{0.95})_2Te_4$) and +$y$-axis (MST), respectively.

Section 5. Crystal anisotropy influence on first and second harmonic Hall signal measurements.

To exclude the crystalline-dependent effect, we have fabricated the crystal-dependent cross devices whose current direction along $\gamma = 0°$, $30°$, $60°$, $75°$ and $90°$ (γ is the angle between the current direction and in-plane crystal axis, displayed in Fig. S5A) on the same MBST sample ($x = 0.95$), shown in Fig. S5B. Then, we carried out the first and second harmonic measurement on γ angle-dependent crossbar devices at T = 1.6 K, the R_{xy} and $R_{xy}^{2\omega}$ results (Figs. S5C-D) displayed that there is no clear anisotropic behavior among different angles which could exclude the possible crystal-related component in first and second harmonic results.

Section 6. Derivation of the first and second harmonic Hall resistances.

As introduced in the manuscript, temperature-dependent magneto-transport measurements were performed using the standard lock-in technique, where a fixed AC current $I = I_0\ sin\omega t$ (*i.e.*, whose amplitude I_0 is kept constant, and ω is the lock-in frequency) was applied along the *x*-direction and the perpendicular magnetic field $\mu_0 H$ was varied between −10 T and +10 T. In this context, the induced effective Oersted field is given by $H_{\text{eff}} = H_I\ sin\omega t$. Along with the applied magnetic field $\mu_0 H$, the transverse Hall resistance R_{xy} can be expressed as*(52)*:

$$R_{xy}(\mu_0 H) = R_{xy}(\mu_0 H + H_I sin\omega t) \approx R_{xy}(\mu_0 H) + \frac{\partial R_{xy}(\mu_0 H)}{\partial(\mu_0 H)} H_I sin\omega t \qquad \text{(S1)}$$

Correspondingly, the measured read-out Hall voltage is given by $V_{xy} = R_{xy}(\mu_0 H) I_0\ sin\omega t$. Finally, by substituting $R_{xy}(\mu_0 H)$ into V_{xy}, we can obtain:

$$V_{xy} = I_0 R_{xy}(\mu_0 H) sin\omega t - \frac{1}{2}\frac{\partial R_{xy}(\mu_0 H)}{\partial(\mu_0 H)} H_I I_0 cos2\omega t + \frac{1}{2}\frac{\partial R_{xy}(\mu_0 H)}{\partial(\mu_0 H)} H_I I_0 \qquad \text{(S2)}$$

where the first term represents the first-order harmonic voltage (*i.e.*, in reference to the static anomalous Hall resistance), while the second term corresponds to the second-order harmonic voltage, which contains information regarding the resistance change due to the change of the magnetic field. Experimentally, after applying the input AC current, the first and second harmonic Hall resistances were recorded using lock-in amplifiers (Stanford Research Systems Model SR830) with the lock-in frequency ranging from 11 to 110 Hz, and the current amplitudes were chosen as 1 μA and 100 μA for the first-harmonic and second-harmonic measurements, respectively.

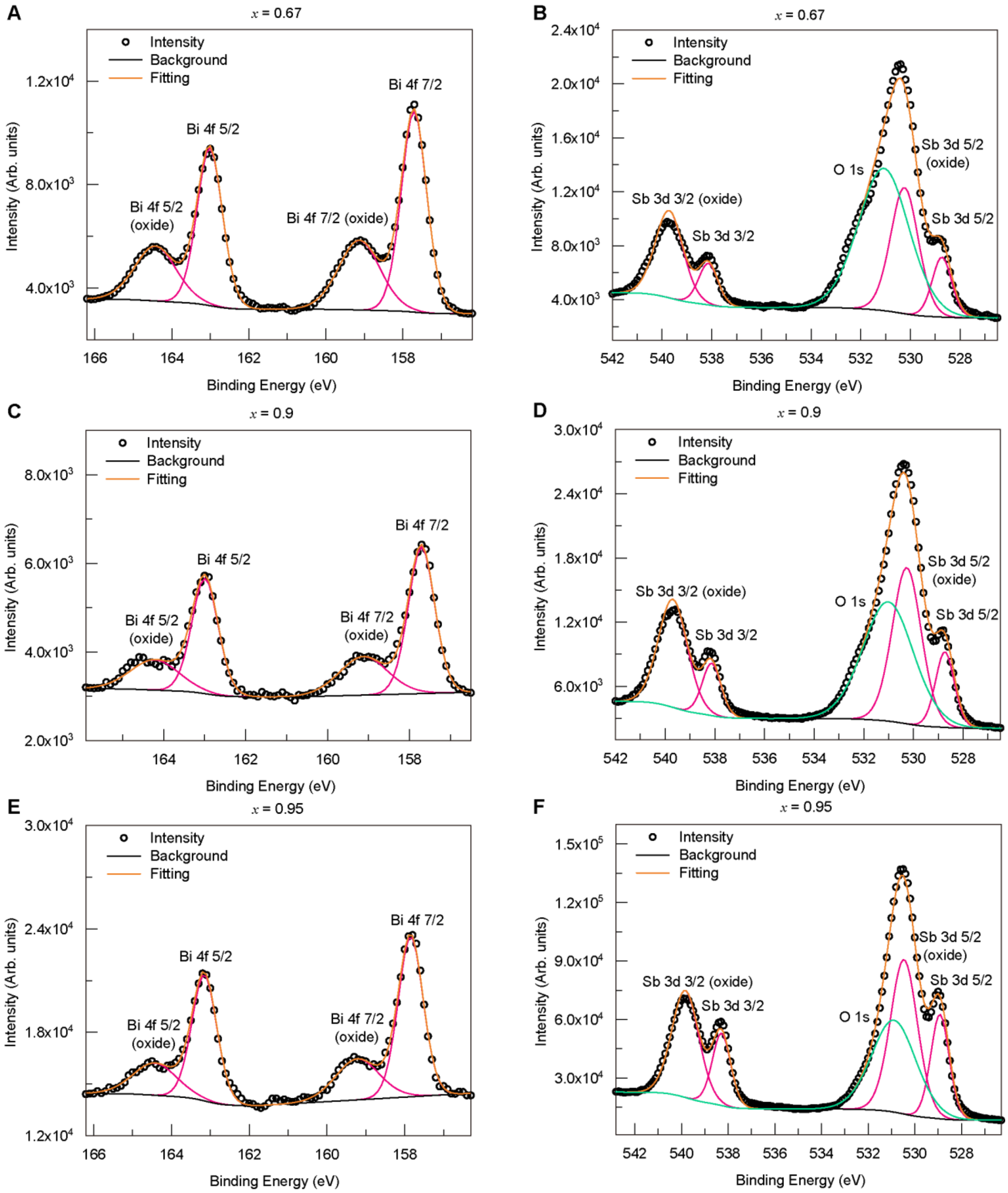

Figure S1. High-resolution XPS spectra of Bi (4*f*) and Sb (3*d*) in the MBE-grown 5 SL $Mn(Bi_{0.33}Sb_{0.67})_2Te_4$ (A-B), $Mn(Bi_{0.1}Sb_{0.9})_2Te_4$ (C-D), and $Mn(Bi_{0.05}Sb_{0.95})_2Te_4$ (E-F) samples.

Nominal x	x (BFM)	x (XPS)
0.67	0.67	0.64
0.9	0.9	0.88
0.95	0.95	0.94

Table S1 Calibration of the Sb fraction x by BFM and XPS measurements.

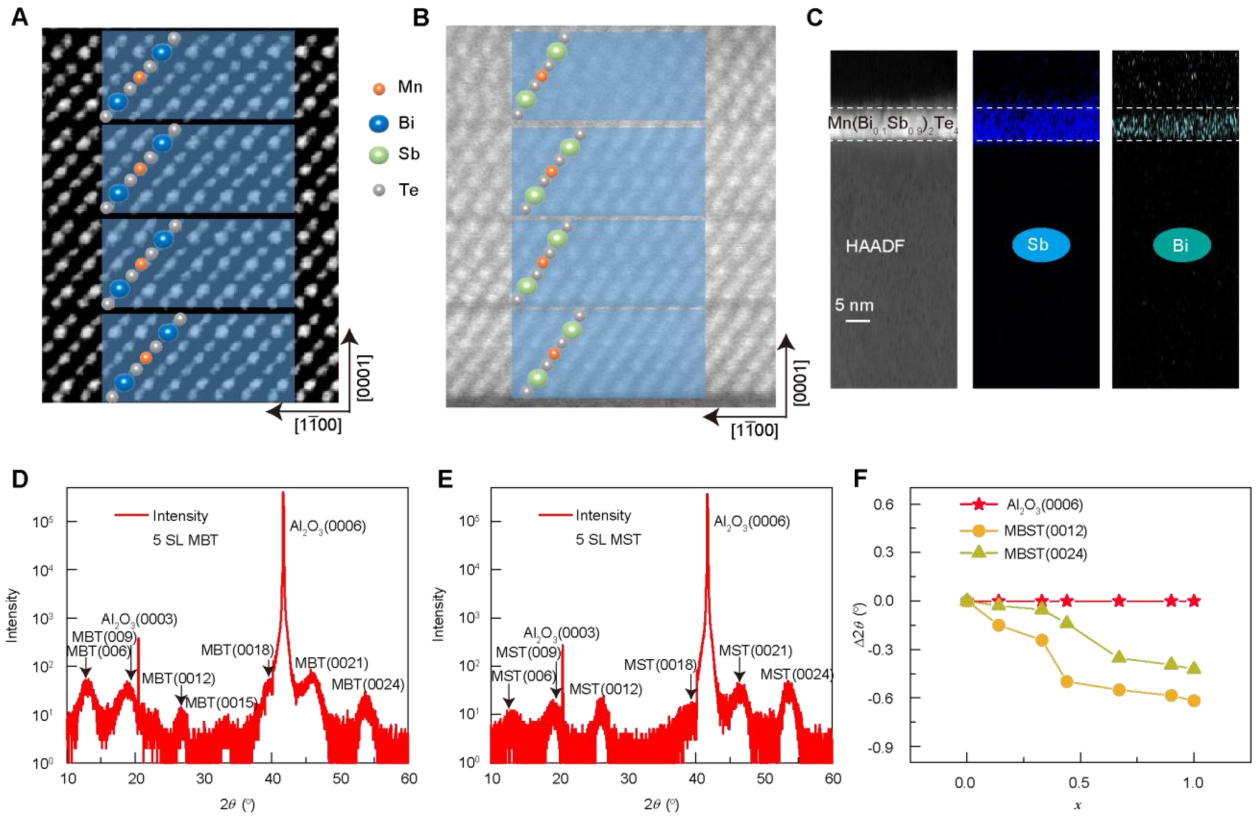

Figure S2. HR-STEM and XRD characterizations of the MBE-grown 5 SL MBST thin films. **A,B,** Cross-sectional HR-STEM image of the $MnBi_2Te_4$ (**A**) (re-captured from Fig. 1C) and $MnSb_2Te_4$ (**B**) thin films grown on $Al_2O_3(0001)$ substrates. **C,** Corresponding EDS maps of the 5 SL $Mn(Bi_{0.1}Sb_{0.9})_2Te_4$ thin film confirm the uniform distribution of both the Bi and Sb atoms. **D,E,** XRD spectra of the 5 SL MBT and MST thin films grown on $Al_2O_3(0001)$ substrates, respectively. **F,** Shift of the XRD peak position as a function of the Sb content x.

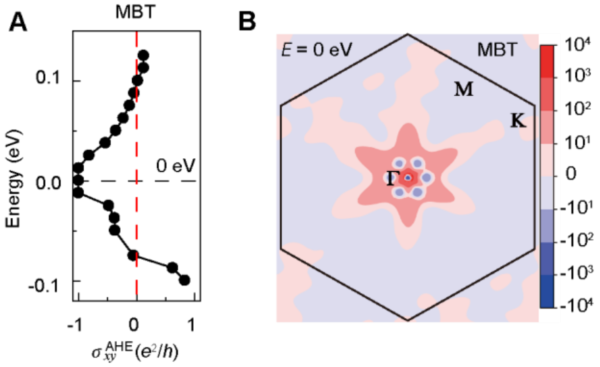

Figure S3. The calculated AHC and three-fold symmetric Berry curvature diagram of MBT. A, Calculated intrinsic anomalous conductance as a function of the chemical potential. **B,** Corresponding Berry curvature distribution for a 5 SL MBT thin film.

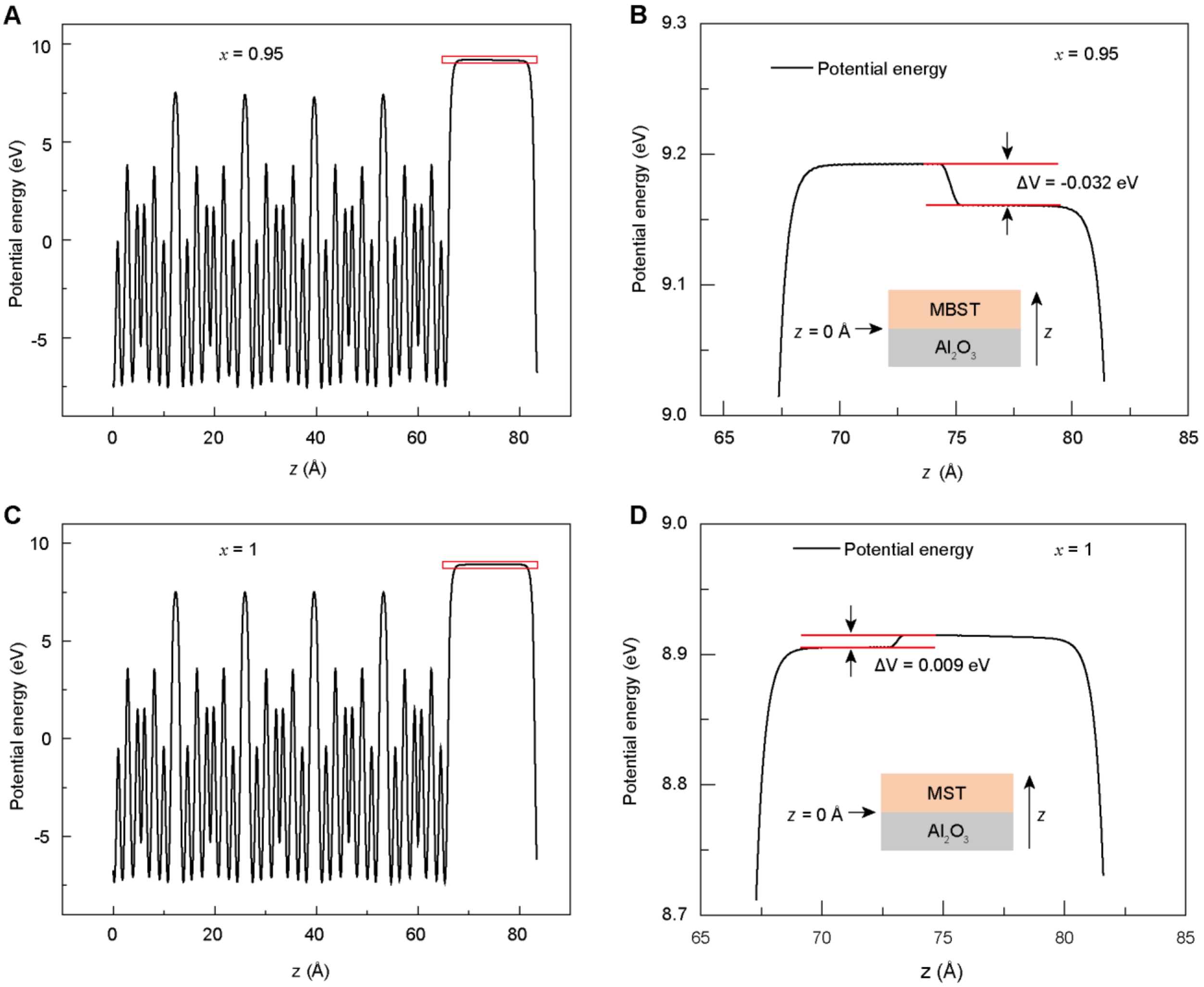

Figure S4. Spatial electrostatic potential distributions of the 5 SL MBST (A,B, *x* = 0.95 and C,D, *x* = 1) thin films. The gradient of the electrostatic potential at the top surface is negative for the $Mn(Bi_{0.05}Sb_{0.95})_2Te_4$ sample, whereas it becomes positive for the $MnSb_2Te_4$ case.

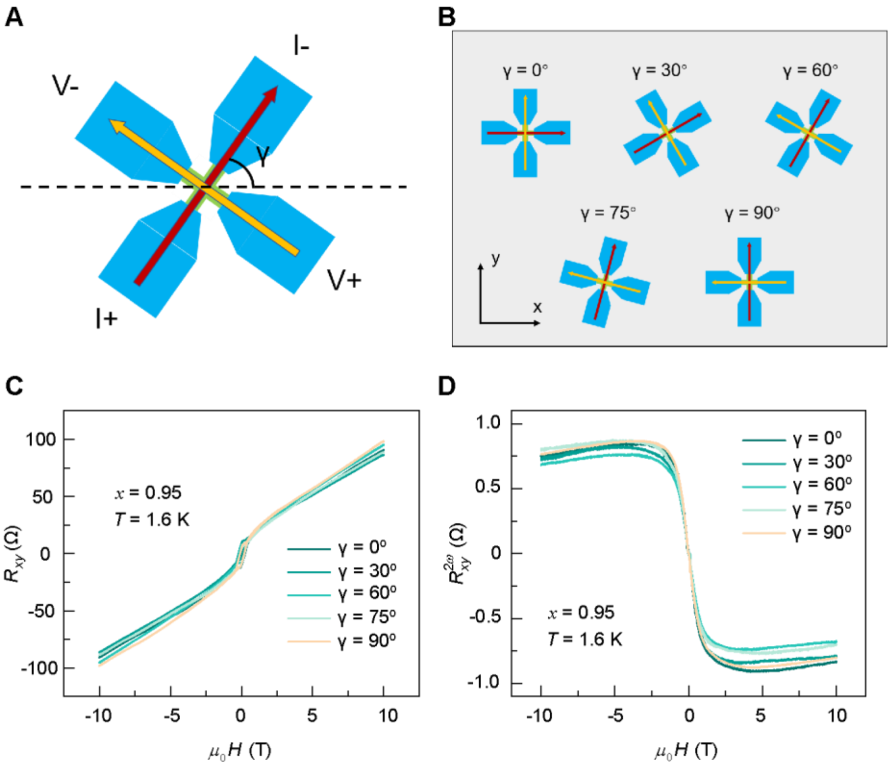

Figure S5. Crystallographic-dependent first and second harmonic Hall measurements on the 5 SL $Mn(Bi_{0.05}Sb_{0.95})_2Te_4$ sample. A,B, Schematics of the cross-bar device structures on the same MBST wafer. γ is defined as the angle between the current direction and in-plane crystal axis of $[11\bar{2}]$. **C,D,** Comparisons of magnetic-field dependent R_{xy} and $R_{xy}^{2\omega}$ at T = 1.6 K.

REFERENCES AND NOTES

1. J. Sinova, S. O. Valenzuela, J. Wunderlich, C. Back, T. Jungwirth, Spin Hall effects. *Rev. Mod. Phys.* **87**, 1213 (2015).

2. M. I. Dyakonov, V. I. Perel, Current-induced spin orientation of electrons in semiconductors. *Phys. Lett. A* **35**, 459–460 (1971).

3. J. Sinova, D. Culcer, Q. Niu, N. Sinitsyn, T. Jungwirth, A. H. MacDonald, Universal intrinsic spin Hall effect. *Phys. Rev. Lett.* **92**, 126603 (2004).

4. S. Murakami, N. Nagaosa, S.-C. Zhang, Dissipationless quantum spin current at room temperature. *Science* **301**, 1348–1351 (2003).

5. A. Manchon, H. C. Koo, J. Nitta, S. M. Frolov, R. A. Duine, New perspectives for Rashba spin–Orbit coupling. *Nat. Mater.* **14**, 871–882 (2015).

6. V. M. Edelstein, Spin polarization of conduction electrons induced by electric current in two-dimensional asymmetric electron systems. *Solid State Commun.* **73**, 233–235 (1990).

7. M. Cubukcu, O. Boulle, N. Mikuszeit, C. Hamelin, T. Brächer, N. Lamard, M.-C. Cyrille, L. Buda-Prejbeanu, K. Garello, I. M. Miron, O. Klein, G. de Loubens, V. V. Naletov, J. Langer, B. Ocker, P. Gambardella, G. Gaudin, Ultra-fast perpendicular spin–orbit torque MRAM. *IEEE Trans. Magn.* **54**, 1–4 (2018).

8. S. Parkin, S.-H. Yang, Memory on the racetrack. *Nat. Nanotechnol.* **10**, 195–198 (2015).

9. Z. Luo, A. Hrabec, T. P. Dao, G. Sala, S. Finizio, J. Feng, S. Mayr, J. Raabe, P. Gambardella, L. J. Heyderman, Current-driven magnetic domain-wall logic. *Nature* **579**, 214–218 (2020).

10. X. Wang, C. Wan, W. Kong, X. Zhang, Y. Xing, C. Fang, B. Tao, W. Yang, L. Huang, H. Wu, M. Irfan, X. Han, Field-free programmable spin logics via chirality-reversible spin–Orbit torque switching. *Adv. Mater.* **30**, 1801318 (2018).

11. S. Fukami, C. Zhang, S. DuttaGupta, A. Kurenkov, H. Ohno, Magnetization switching by spin–Orbit torque in an antiferromagnet–ferromagnet bilayer system. *Nat. Mater.* **15**, 535–541 (2016).

12. A. Kurenkov, S. DuttaGupta, C. Zhang, S. Fukami, Y. Horio, H. Ohno, Artificial neuron and synapse realized in an antiferromagnet/ferromagnet heterostructure using dynamics of spin–orbit torque switching. *Adv. Mater.* **31**, e1900636 (2019).

13. K. Garello, I. M. Miron, C. O. Avci, F. Freimuth, Y. Mokrousov, S. Blügel, S. Auffret, O. Boulle, G. Gaudin, P. Gambardella, Symmetry and magnitude of spin–orbit torques in ferromagnetic heterostructures. *Nat. Nanotechnol.* **8**, 587–593 (2013).

14. C.-F. Pai, L. Liu, Y. Li, H. Tseng, D. Ralph, R. Buhrman, Spin transfer torque devices utilizing the giant spin Hall effect of tungsten. *Appl. Phys. Lett.* **101**, 122404 (2012).

15. L. Liu, C.-F. Pai, Y. Li, H. W. Tseng, D. C. Ralph, R. A. Buhrman, Spin-torque switching with the giant spin Hall effect of tantalum. *Science* **336**, 555–558 (2012).

16. R. Ramaswamy, X. Qiu, T. Dutta, S. D. Pollard, H. Yang, Hf thickness dependence of spin-orbit torques in Hf/CoFeB/MgO heterostructures. *Appl. Phys. Lett.* **108**, 202406 (2016).

17. Q. L. He, T. L. Hughes, N. P. Armitage, Y. Tokura, K. L. Wang, Topological spintronics and magnetoelectronics. *Nat. Mater.* **21**, 15–23 (2022).

18. Y. Tokura, K. Yasuda, A. Tsukazaki, Magnetic topological insulators. *Nat. Rev. Phys.* **1**, 126–143 (2019).

19. Y. Fan, P. Upadhyaya, X. Kou, M. Lang, S. Takei, Z. Wang, J. Tang, L. He, L.-T. Chang, M. Montazeri, G. Yu, W. Jiang, T. Nie, R. N. Schwartz, Y. Tserkovnyak, K. L. Wang, Magnetization switching through giant spin–orbit torque in a magnetically doped topological insulator heterostructure. *Nat. Mater.* **13**, 699–704 (2014).

20. Y. Fan, X. Kou, P. Upadhyaya, Q. Shao, L. Pan, M. Lang, X. Che, J. Tang, M. Montazeri, K. Murata, L. T. Chang, M. Akyol, G. Yu, T. Nie, K. L. Wong, J. Liu, Y. Wang, Y. Tserkovnyak, K.

L. Wang, Electric-field control of spin–Orbit torque in a magnetically doped topological insulator. *Nat. Nanotechnol.* **11**, 352–359 (2016).

21. K. Yasuda, A. Tsukazaki, R. Yoshimi, K. Kondou, K. Takahashi, Y. Otani, M. Kawasaki, Y. Tokura, Current-nonlinear Hall effect and spin-orbit torque magnetization switching in a magnetic topological insulator. *Phys. Rev. Lett.* **119**, 137204 (2017).

22. J. Han, A. Richardella, S. A. Siddiqui, J. Finley, N. Samarth, L. Liu, Room-temperature spin-orbit torque switching induced by a topological insulator. *Phys. Rev. Lett.* **119**, 077702 (2017).

23. L. Tian, Y. Liu, W. Meng, X. Zhang, X. Dai, G. Liu, Spin–orbit coupling-determined topological phase: Topological insulator and quadratic Dirac semimetals. *J. Phys. Chem. Lett.* **11**, 10340–10347 (2020).

24. N. Nagaosa, J. Sinova, S. Onoda, A. H. MacDonald, N. P. Ong, Anomalous hall effect. *Rev. Mod. Phys.* **82**, 1539–1592 (2010).

25. Z. Zhang, N. Wang, N. Cao, A. Wang, X. Zhou, K. Watanabe, T. Taniguchi, B. Yan, W.-B. Gao, Controlled large non-reciprocal charge transport in an intrinsic magnetic topological insulator $MnBi_2Te_4$. *Nat. Commun.* **13**, 6191 (2022).

26. K. Yasuda, T. Morimoto, R. Yoshimi, M. Mogi, A. Tsukazaki, M. Kawamura, K. S. Takahashi, M. Kawasaki, N. Nagaosa, Y. Tokura, Large non-reciprocal charge transport mediated by quantum anomalous Hall edge states. *Nat. Nanotechnol.* **15**, 831–835 (2020).

27. M. M. Otrokov, T. V. Menshchikova, M. G. Vergniory, I. P. Rusinov, A. Y. Vyazovskaya, Y. M. Koroteev, G. Bihlmayer, A. Ernst, P. M. Echenique, A. Arnau, Highly-ordered wide bandgap materials for quantized anomalous Hall and magnetoelectric effects. *2D Mater.* **4**, 025082 (2017).

28. B. Chen, F. Fei, D. Zhang, B. Zhang, W. Liu, S. Zhang, P. Wang, B. Wei, Y. Zhang, Z. Zuo, J. Guo, Q. Liu, Z. Wang, X. Wu, J. Zong, X. Xie, W. Chen, Z. Sun, S. Wang, Y. Zhang, M. Zhang, X. Wang, F. Song, H. Zhang, D. Shen, B. Wang, Intrinsic magnetic topological insulator phases in the Sb doped $MnBi_2Te_4$ bulks and thin flakes. *Nat. Commun.* **10**, 4469 (2019).

29. S. H. Lee, D. Graf, L. Min, Y. Zhu, H. Yi, S. Ciocys, Y. Wang, E. S. Choi, R. Basnet, A. Fereidouni, A. Wegner, Y. F. Zhao, K. Verlinde, J. He, R. Redwing, V. Gopalan, H. O. H. Churchill, A. Lanzara, N. Samarth, C. Z. Chang, J. Hu, Z. Q. Mao, Evidence for a magnetic-field-induced ideal type-II Weyl state in antiferromagnetic topological insulator $Mn(Bi_{1-x}Sb_x)_2Te_4$. *Phys. Rev. X* **11**, 031032 (2021).

30. P. Chen, Q. Yao, J. Xu, Q. Sun, A. J. Grutter, P. Quarterman, P. P. Balakrishnan, C. J. Kinane, A. J. Caruana, S. Langridge, Tailoring the magnetic exchange interaction in $MnBi_2Te_4$ superlattices via the intercalation of ferromagnetic layers. *Nat. Electron.* **6**, 18–27 (2023).

31. J.-Q. Yan, S. Okamoto, M. A. McGuire, A. F. May, R. J. McQueeney, B. C. Sales, Evolution of structural, magnetic, and transport properties in $MnBi_{2-x}Sb_xTe_4$. *Phys. Rev. B* **100**, 104409 (2019).

32. R. Watanabe, R. Yoshimi, M. Kawamura, Y. Kaneko, K. S. Takahashi, A. Tsukazaki, M. Kawasaki, Y. Tokura, Enhancement of anomalous Hall effect in epitaxial thin films of intrinsic magnetic topological insulator $MnBi_2Te_4$ with Fermi-level tuning. *Appl. Phys. Lett.* **120**, 031901 (2022).

33. D. Xiao, M.-C. Chang, Q. Niu, Berry phase effects on electronic properties. *Rev. Mod. Phys.* **82**, 1959 (2010).

34. X. Chen, S. Shi, G. Shi, X. Fan, C. Song, X. Zhou, H. Bai, L. Liao, Y. Zhou, H. Zhang, A. Li, Y. Chen, X. Han, S. Jiang, Z. Zhu, H. Wu, X. Wang, D. Xue, H. Yang, F. Pan, Observation of the antiferromagnetic spin Hall effect. *Nat. Mater.* **20**, 800–804 (2021).

35. C. O. Avci, K. Garello, A. Ghosh, M. Gabureac, S. F. Alvarado, P. Gambardella, Unidirectional spin Hall magnetoresistance in ferromagnet/normal metal bilayers. *Nat. Phys.* **11**, 570–575 (2015).

36. G. Liu, X. Wang, Z. Luan, L. Zhou, S. Xia, B. Yang, Y. Tian, G.-h. Guo, J. Du, D. Wu, Magnonic unidirectional spin Hall magnetoresistance in a heavy-metal–ferromagnetic-insulator bilayer. *Phys. Rev. Lett.* **127**, 207206 (2021).

37. Y. Lv, J. Kally, D. Zhang, J. S. Lee, M. Jamali, N. Samarth, J.-P. Wang, Unidirectional spin-Hall and Rashba−Edelstein magnetoresistance in topological insulator-ferromagnet layer heterostructures. *Nat. Commun.* **9**, 111 (2018).

38. S.-H. C. Baek, V. P. Amin, Y.-W. Oh, G. Go, S.-J. Lee, G.-H. Lee, K.-J. Kim, M. D. Stiles, B.-G. Park, K.-J. Lee, Spin currents and spin–orbit torques in ferromagnetic trilayers. *Nat. Mater.* **17**, 509–513 (2018).

39. H. Masuda, T. Seki, J.-I. Ohe, Y. Nii, H. Masuda, K. Takanashi, Y. Onose, Room temperature chirality switching and detection in a helimagnetic $MnAu_2$ thin film. *Nat. Commun.* **15**, 1999 (2024).

40. G. Kresse, J. Furthmüller, Efficient iterative schemes for ab initio total-energy calculations using a plane-wave basis set. *Phys. Rev. B Condens. Matter* **54**, 11169–11186 (1996).

41. J. P. Perdew, K. Burke, M. Ernzerhof, Generalized gradient approximation made simple. *Phys. Rev. Lett.* **77**, 3865–3868 (1996).

42. V. I. Anisimov, J. Zaanen, O. K. Andersen, Band theory and Mott insulators: Hubbard U instead of Stoner I. *Phys. Rev. B Condens. Matter* **44**, 943 –954 (1991).

43. H. J. Monkhorst, J. D. Pack, Special points for Brillouin-zone integrations. *Phys. Rev. B* **13**, 5188 (1976).

44. S. Grimme, J. Antony, S. Ehrlich, H. Krieg, A consistent and accurate ab initio parametrization of density functional dispersion correction (DFT-D) for the 94 elements H-Pu. *J. Chem. Phys.* **132**, 154104 (2010).

45. A. A. Mostofi, J. R. Yates, Y.-S. Lee, I. Souza, D. Vanderbilt, N. Marzari, wannier90: A tool for obtaining maximally-localised Wannier functions. *Comput. Phys. Commun.* **178**, 685–699 (2008).

46. Q. Wu, S. Zhang, H.-F. Song, M. Troyer, A. A. Soluyanov, WannierTools: An open-source software package for novel topological materials. *Comput. Phys. Commun.* **224**, 405–416 (2018).

47. H. Li, S. Liu, C. Liu, J. Zhang, Y. Xu, R. Yu, Y. Wu, Y. Zhang, S. Fan, Antiferromagnetic topological insulator $MnBi_2Te_4$: Synthesis and magnetic properties. *Phys. Chem. Chem. Phys.* **22**, 556–563 (2020).

48. H. Li, Y. Li, Y. Lian, W. Xie, L. Chen, J. Zhang, Y. Wu, S. Fan, Glassy magnetic ground state in layered compound $MnSb_2Te_4$. *Sci. China Mater.* **65**, 477–485 (2022).

49. Y. Deng, Y. Yu, M. Z. Shi, Z. Guo, Z. Xu, J. Wang, X. H. Chen, Y. Zhang, Quantum anomalous Hall effect in intrinsic magnetic topological insulator $MnBi_2Te_4$. *Science* **367**, 895–900 (2020).

50. C. Liu, Y. Wang, H. Li, Y. Wu, Y. Li, J. Li, K. He, Y. Xu, J. Zhang, Y. Wang, Robust axion insulator and Chern insulator phases in a two-dimensional antiferromagnetic topological insulator. *Nat. Mater.* **19**, 522–527 (2020).

51. X. Kou, Y. Fan, K. L. Wang, Review of quantum Hall trio. *J. Phys. Chem. Solid* **128**, 2–23 (2019).

52. C. Song, R. Zhang, L. Liao, Y. Zhou, X. Zhou, R. Chen, Y. You, X. Chen, F. Pan, Spin-orbit torques: Materials, mechanisms, performances, and potential applications. *Prog.Mater. Sci.* **118**, 100761 (2021).
